# Constitutive IFNα Protein Production in Bats

**DOI:** 10.3389/fimmu.2021.735866

**Published:** 2021-11-01

**Authors:** Vincent Bondet, Maxime Le Baut, Sophie Le Poder, Alexis Lécu, Thierry Petit, Rudy Wedlarski, Darragh Duffy, Delphine Le Roux

**Affiliations:** ^1^ Translational Immunology Lab, Institut Pasteur, Université de Paris, Paris, France; ^2^ BioPôle Alfort, Ecole Nationale Vétérinaire d’Alfort, Maisons-Alfort, France; ^3^ Anses, INRAE, Ecole Nationale Vétérinaire d’Alfort, UMR VIROLOGIE, Laboratoire de Santé Animale, Maisons-Alfort, France; ^4^ Parc Zoologique de Paris, Muséum National d’Histoire Naturelle, Paris, France; ^5^ Parc Zoologique de La Palmyre, Les Mathes, France; ^6^ Bioparc Zoo de Doué La Fontaine, Doué-la-Fontaine, France; ^7^ Anses, INRAE, Ecole Nationale Vétérinaire d’Alfort, UMR BIPAR, Laboratoire de Santé Animale, Maisons-Alfort, France

**Keywords:** Chiroptera, bats, type I IFN, Simoa digital ELISA, protein levels, antiviral immunity

## Abstract

Bats are the only mammals with self-powered flight and account for 20% of all extant mammalian diversity. In addition, they harbor many emerging and reemerging viruses, including multiple coronaviruses, several of which are highly pathogenic in other mammals, but cause no disease in bats. How this symbiotic relationship between bats and viruses exists is not yet fully understood. Existing evidence supports a specific role for the innate immune system, in particular type I interferon (IFN) responses, a major component of antiviral immunity. Previous studies in bats have shown that components of the IFN pathway are constitutively activated at the transcriptional level. In this study, we tested the hypothesis that the type I IFN response in bats is also constitutively activated at the protein level. For this, we utilized highly sensitive Single Molecule (Simoa) digital ELISA assays, previously developed for humans that we adapted to bat samples. We prospectively sampled four non-native chiroptera species from French zoos. We identified a constitutive expression of IFNα protein in the circulation of healthy bats, and concentrations that are physiologically active in humans. Expression levels differed according to the species examined, but were not associated with age, sex, or health status suggesting constitutive IFNα protein expression independent of disease. These results confirm a unique IFN response in bat species that may explain their ability to coexist with multiple viruses in the absence of pathology. These results may help to manage potential zoonotic viral reservoirs and potentially identify new anti-viral strategies.

## Introduction

With more than 1,200 species, representing about 20% of the total diversity of Mammals, bats are among the most abundant, diverse and geographically dispersed vertebrates on the planet. They are the only mammals capable of active flight and present numerous anatomical variations (1–4). Bats also act as reservoirs for a multitude of viruses, some recognized as highly pathogenic to humans and animals (5–10). Moreover, bats have been shown to be involved in the emergence and re-emergence of numerous highly pathogenic zoonotic viruses such as Rhabdoviridae, Paramyxoviridae (Nipah and Hendra viruses), Filoviridae (Ebola and Marburg viruses), and Coronaviridae (11–19) for which they are suspected to be involved in the emergence of the original SARS-CoV-2 viral strain (20). Moreover, most of the bats experimentally infected with viral doses of Hepinaviruses or Lyssaviruses, which are lethal to other mammals, did not show apparent clinical signs (21–23). It is likely that viruses and their bat hosts have undergone a long process of co-evolution that began several million years ago with the appearance of the first Chiroptera (9, 24, 25). These mechanisms, along with their specific characteristics including longevity, migratory activity, active flight, and population density, may have shaped both the bat immune system and their ability to thwart host responses to viruses, resulting in a balance between persistent infection and absence of pathophysiology (5, 6, 10, 26, 27). One hypothesis is that bats are able to control viral replication through the existence of specific innate antiviral mechanisms (28). Among them, the production of IFN is known to be the first line of defence against viral infections (29) and there is evidence of a strong constitutive genomic expression of type I IFN, mainly of IFNα, in at least two species of Chiroptera (*P. alecto* and *C. brachyotis*) (30). This difference is unique since it has not been observed in other mammals, however, it remains to be confirmed whether this constitutive expression occurs at the protein level.

We have previously used ultra-sensitive Single Molecule Array (Simoa) digital ELISA to measure IFNα protein in the serum of human patients with autoimmune diseases or viral infection whose levels were previously undetectable with conventional ELISA techniques (31). This ultra-sensitive technique is therefore capable of measuring cytokines at very low concentrations in biological fluids, which were previously only measured indirectly by detection of downstream gene induction. Thus, the measurement of IFNα protein in Chiroptera may confirm the hypothesis raised by Zhou et al. which is based solely on mRNA measurements and not on direct protein quantification (30). Indeed, constitutively expressed type I IFN mRNA in bats could represent a “ready to use” pool during viral infection, or it could be directly translated into protein resulting in high blood concentrations and viral protection. In this study, we quantified bat IFNα2 protein using ultra-sensitive digital ELISA on plasma from four species of captive bats: *P. rodricensis*, *P. lylei*, *R. aegyptiacus* and *E. helvum*. We also correlated the IFNα protein expression to the corresponding mRNA levels. This study provides new evidence of how the unique immune system of bats may control viruses in the absence of disease and in doing so constitute a constant viral reservoir for zoonotic transmission and potential new pandemics.

## Materials and Methods

### Bat Cohort and Sampling

Four bat species from four French zoos were sampled, during their annual sanitary examination, by the resident veterinary doctor. 0.2 to 0.5 mL of blood was drawn from different veins depending on the species and collected in an EDTA containing tube using a 1mL syringe and a 25G x 5/8 needle (all from Beckton Dickinson, France). Under general anesthesia (O_2_ at 1.5L/min and 5% isoflurane for induction and 2% to maintain the anesthesia) blood of *P. rodricensis* and *R. aegyptiacus* was taken from the medial vein and jugular vein, respectively. *E. helvum* and *P. lylei* were vigilant during blood sampling, which was done from the medial vein for both species. The demographic characteristics and the origins of this cohort are indicated in **Table 1**. Blood was then split into PAXGene tubes (PreAnalytix GmbH, Qiagen, France) for RNA extraction and RT-qPCR analysis, and Eppendorf tubes to obtain plasma. PAXGene tubes were stored at -20°C until extraction, while Eppendorf tubes were centrifuged at 2500rpm for 10min. Plasma was then removed and stored at -80°C until Simoa analysis. Human plasma samples measured for IFNα2 from previously published studies were included for comparison including healthy controls (31), patients with systemic lupus erythematosus (SLE) and acute dengue viral infection (32).

**Table 1:** Demographic characteristics and origins of the bat cohort.

Number of individuals	Gender, female	Species (n, %)	Origin
108	36 (43%)	*Pteropus rodricensis:* 21 (19%)	Parc Zoologique de La Palmyre
		*Rousettus aegyptiacus:* 10 (9.3%)	Parc Zoologique de La Palmyre
		*Eidolon helvum:* 23 (21%)	Parc Zoologique de Paris
		*Pteropus lylei:* 54 (50%)	Bioparc Zoo de Doué La Fontaine

Number of individuals, gender, species and origins for the bat cohort. Data are shown as the n (%).

### Bat Cell Stimulation Assays

Bat epithelial cells from Tb 1 Lu cell line (ATCC, United States) were cultured at 37°C, 5% CO_2_, in complete culture medium composed of MEM Eagle medium with 2 mM Glutamine supplemented with 50 U/mL penicillin, 50 μg/mL streptomycin (all from Lonza, Belgium) and with 10% of decomplemented fetal calf serum (Gibco, Thermo Fisher Scientific, France). Before stimulation, cells were plated in 1mL of complete medium per well of 24 well plates and maintained at 37°C, 5% CO_2_ until they reached 2x10^6^ cells/well. Supernatants were removed and 1mL of complete medium including 500HAU/mL mouse influenza virus (Strain H1N1 A/PR81934), an available and known stimulus for type I interferon response that can be manipulated in P2 facilities, was added to the cells (positive control) or not (unstimulated control). Before stimulation and 1 hour, 3.5 hours, and 23 hours after stimulation, supernatants were sampled and frozen at -80°C for IFNα protein quantification.

### Production of the *Rousettus aegyptiacus* IFNα Protein for ELISA Calibration

The *Rousettus aegyptiacus* IFNα DNA sequence was obtained from a previously published study (33) (**Figure S1A**). Nucleotide bases that correspond to the signal peptide were removed, a start codon, spacers, and codons for a 6His tag and a TEV cleavage site were added in the 5’ termination (**Figure S1B**). The cDNA coding for the recombinant protein was chemically-synthesized with optimization for expression in *Escherichia coli*. The recombinant gene was then introduced in a pT7 expression plasmid under the control of a Lac operator and harboring kanamycin resistance (**Figure S1C**). *E. coli* strains were transformed and kanamycin-resistant clones were selected. After optimization, protein production was done, culturing the selected clones in a Luria–Bertani kanamycin (LBkan) medium at 16°C during 16 hours after induction with 1mM isopropyl β-D-1-thiogalactopyranoside (IPTG). Bacteria were then harvested by centrifugation. The pellet was lysed and the soluble extract was obtained after a second centrifugation. It was aliquoted and stored at -80°C. This soluble extract was directly used at different dilutions as a calibrator for the digital ELISA assay. The total protein level of the soluble extract was quantified using the BCA assay. Briefly, proteins reduce Cu^2+^ to Cu^+^ in an alkaline medium, and the cuprous cation generated is detected by bicinchoninic acid (BCA) forming a blue-to-violet complex that absorbs light at 562nm. The soluble extract was then analysed by SDS-Page, and the bIFNα protein band identified by Western-Blot using the detector antibody of the hIFNα2 digital ELISA assay. Then, its proportion in the total proteins of the extract was estimated using gel densitometry, and the recombinant bIFNα concentration of the soluble extract calculated.

### Sample Preparation for hIFNα2 Digital ELISA Assay

All plasma samples were first thawed and centrifuged at 10.000g, +4°C for 10 minutes to remove debris. Because bats can harbor many viruses, supernatants were treated in a P2 laboratory for viral inactivation using a standard solvent/detergent protocol used for human blood plasma products (34, 35) and described in (36) and in (32). Briefly, samples were treated with Tri-*n*-Butyl Phosphate (T*n*BP) 0.3% (v/v) and Triton X100 (TX100) 1% (v/v) for 2 hours at room temperature. After treatment, T*n*BP was removed by passing the samples through a C18 column (Discovery DSC-18 SPE from Supelco). For digital ELISA assays, inactivated samples and stimulated cell supernatants were diluted in the Detector/Sample Diluent (Quanterix) added with NP40 0,5% (v/v). They were then incubated for one hour at room temperature before analysis. Global dilution factor was generally 1/6 for plasma samples and 1/3 for stimulated cell supernatants depending on the amount of material available and to allow the optimal protein detection.

### hIFNα2 Digital ELISA Assay

The Simoa hIFNα2 assay was developed using the Quanterix Homebrew kit and described in (32). The BMS216C (eBioscience) antibody clone was used as a capture antibody after coating on paramagnetic beads (0.3mg/mL), and the BMS216BK biotinylated antibody clone was used as the detector at a concentration of 0.3ug/mL. The SBG revelation enzyme concentration was 150pM. The assay follows a 2-step ELISA configuration. Two calibrators were used; recombinant human IFNα2c (hIFNα2c) purchased from eBioscience and *Rousettus aegyptiacus* IFNα (bIFNα) produced in *Escherichia coli* for this study. The limit of detection (LOD) was calculated by the mean value of all blank runs + 2SD after log conversion.

### RNA Extraction and IFNα RT-qPCR

Whole blood RNA was extracted manually from PAXGene tubes, following manufacturer’s instructions (Blood RNA extraction kit, Qiagen, France). After extraction, samples were inactivated at 65°C for 5min then stored at -80°C until RT-qPCR. RT-qPCR was done using the qScript XLT One-Step RT-qPCR mix following manufacturer’s instructions (Quanta BioSciences, Inc., United States). Taqman probes (Applied Biosystems, ThermoFisher Scientific, France) and primers (Eurofins, France) for IFN-α1, IFNα2 and IFNα3 were described previously for *P. alecto* bat species in (30) and used here. Probes and primers for the bat GAPDH housekeeping gene were designed using Primer-BLAST from NCBI (https://www.ncbi.nlm.nih.gov/tools/primer-blast/) and are presented in **Table S2**. All data were normalized relative to the housekeeping gene (GAPDH) as indicated. The expression level of the target genes was calculated using the standard curve method and expressed as copy numbers relative to the housekeeping gene.

### Nested RT-qPCR for Pan-Coronaviruses in mRNA From Bat Whole Blood

RT-qPCR for potential coronaviruses in bat whole blood RNA was performed from the mRNA extracted previously and following the protocol previously published in (37).

### Statistical Analyses

GraphPad Prism 8 was used for statistical analysis. Mann-Whitney tests were used to compare two groups such as female and male, or healthy and clinical alterations. ANOVA tests (Kruskal–Wallis) with Dunn’s post testing for multiple comparisons were used to test for differences between multiple bat species. For all analyses, p values less than 0.05 were considered statistically significant, with *p<0.05; **p<0.01; ***p<0.001; ****p<0.0001. Median values were reported on figures. Spearman correlations are used to compare continuous variables such as mRNA level or age and protein production.

### Safety and Ethical Considerations

The bat specimens included in this study are not wild but obtained from zoos where they have been maintained for several generations. Animals were not sampled for the purpose of this study. Instead, blood was taken during routine clinical examination by authorized veterinarians for which no specific ethical requirements were required. However, for biosafety precautions, samples were handled in a P2 biosafety level laboratory where they were treated with a viral inactivation protocol before use, as described above.

### Data Availability

All available data from the bat cohort are shown in **Supplementary Table S1**.

## Results

### Bat IFNα Protein Detection With a hIFNα2 Digital ELISA Assay

**Figure 1:**
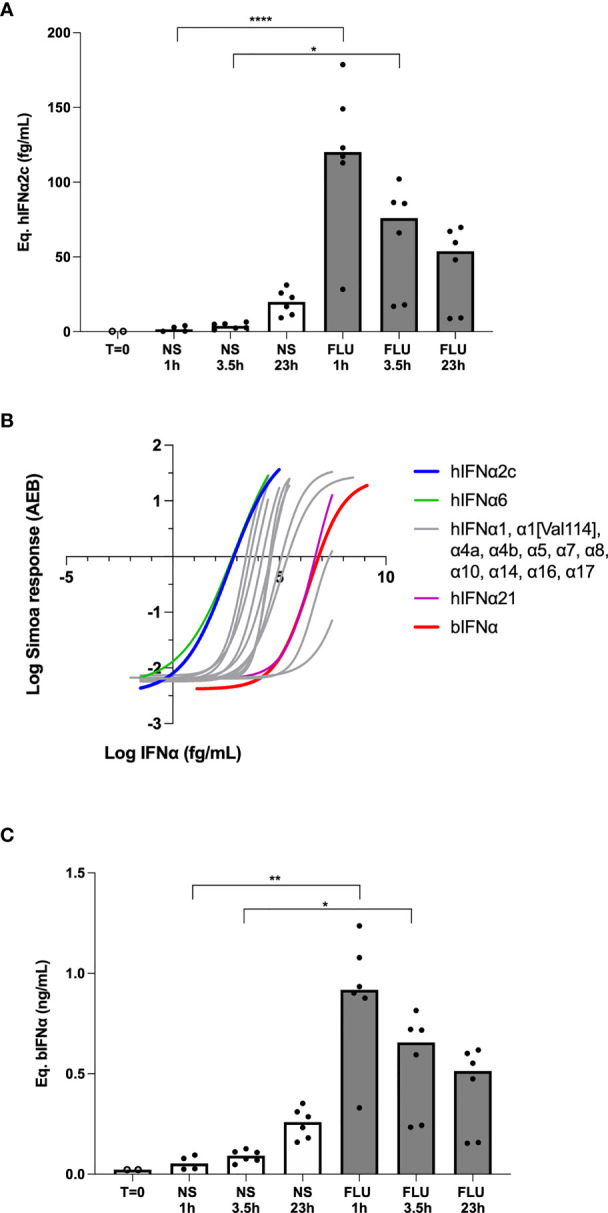
Bat IFNα proteins are detected with a human IFNα2 digital ELISA assay. **(A)** IFNα protein levels expressed as equivalent human IFNα2c concentrations obtained after stimulation of bat epithelial cells Tb 1 Lu with 500HAU/mL mouse influenza virus (FLU) or unstimulated (NS) for 0 to 24 hours at 37°C and 5% CO_2_. **(B)** hIFNα2 digital ELISA assay response (AEB) as a function of the IFNα concentration for the *Rousettus aegyptiacus* IFNα calibrator (bIFNα) produced in *Escherichia coli* (red) in comparison with the human IFNα2c calibrator (blue) and the 12 other human IFNα subtypes. **(C)** IFNα protein levels expressed as equivalent bIFNα concentrations obtained after stimulation of bat epithelial cells as described previously. Box plots represent median and individual values represented by dots are reported on figures, **(A, C)** representing pooled results from 3 independent experiments. Kruskal–Wallis test with Dunn’s post testing for multiple comparisons was used, *p < 0.05, **p < 0.01, ****p < 0.0001.

Anti-bat IFNα antibodies are not commercially available for the development of bat-specific IFNα ELISA. However, given the ultra-sensitivity of human IFNα digital ELISA which detects protein at attomolar concentrations (31), and potential cross-species reactivity, we hypothesized that our existing human assay could also detect bat IFNα. As a first proof of concept, we stimulated a bat lung epithelial cell line with influenza virus (Strain H1NI A/PR81934) and tested the recovered supernatant with a human IFNα2 digital ELISA. We observed a significant induction of IFNα proteins at 1hr and 3.5 hrs as compared to the unstimulated control (**Figure 1A**).

These initial results were extrapolated from a standard curve of a human recombinant IFNα2 protein. To better adapt our assay to bat species, we produced a recombinant *Rousettus aegyptiacus* IFNα protein (bIFNα) from *Escherichia coli* competent bacteria. SDS-Page analysis of the soluble and insoluble extracts obtained from the bacteria pellet showed that bIFNα was mainly produced as an insoluble form even at low induction temperature (**Figure S1D**). Comparing profiles before and after induction of the protein expression, SDS-Page analysis showed that the unique bIFNα band appeared alone at this mass (**Figure S1D**). Western-Blot analysis of the soluble fraction after induction at 16°C using the hIFNα2 assay detection antibody revealed that the protein was expressed in a single band at the expected molecular weight (**Figure S1D**). The purification from the soluble extract failed: the bIFNα protein was not selected at the expected molecular weight (**Figure S1E**) and the western-Blot analysis revealed no affinity at the purified molecular weight (**Figure S1D**). A possible explanation is that a high proportion of the protein was not properly folded, in relation with its insolubility. The purification from the insoluble extract succeeded, but the renaturation of the protein failed (**Figure S1F**). So we used the soluble extract itself as a calibrator after quantification of bIFNα. Global protein quantification of the soluble extract was done using the BCA assay, and the bIFNα protein concentration in the soluble extract was estimated after gel densitometry for potential use as a digital ELISA calibrator.

To explore the ability of the hIFNα2 digital ELISA assay designed for the quantification of human interferons, to quantify bat IFNα species, we compared the responses of the assay to bIFNα protein and all 13 human IFNα subtypes (**Figure 1B**). As expected, the assay revealed a weaker response for bIFNα in comparison with hIFNα2c. However, the affinity of the human mAb for the bat protein was comparable to the human subtypes, with bIFNα and hIFNα21 showing very similar affinities, and two human species showing weaker responses (**Figure 1B**). Using the bIFNα protein as the calibrator, we re-calculated the cellular response after *in vitro* influenza stimulation and observed similar results with the highest concentrations present after 1hr of influenza stimulation (**Figure 1C**). The only difference observed was related to the scale of these results, due to the lower affinity of the mAb for the bIFNa2 calibrator.

To better understand the cross-species reactivity we compared the sensitivities of the 13 IFNα subtypes as previously described, the 5 other human IFNβ, IFNΛ1, IFNΛ2, IFNω and IFNγ, and the 5 mouse IFNα1, IFNα3, IFNα4, IFNα11 and IFNα13, with their available online sequences in the UniProtKB database (www.uniprot.org) after alignment using the CLUSTALW software (www.genome.jp/tools-bin/clustalw). We also considered the fact that epitopes must be accessible to the antibodies and so studied the IFNα2, IFNα14 and IFNω three-dimensional structures published online in the PDB database (www.rcsb.org) after spacial alignment using the PyMOL software (www.pymol.org). This analysis suggests that the epitope recognized by the hIFNα2 mAb could be the ^110^LMKED sequence in the human IFNα2 molecule. *Rousettus aegyptiacus* IFNα and *Pteropus rodricensis* IFNα1, IFNα2 and IFNα3 protein sequences were previously described by Omatsu et al. (33) and Zhou et al. (30) respectively. Alignment of these molecules showed that the corresponding amino-acids in the hIFNα2 assay epitope position are LMNED for the 3 IFNα species from *Pteropus* and LLDED for *Rousettus* (**Figure S2A**). The LMNED sequence also appears in human IFNα16 and IFNα17, two species for which the hIFNα2 assay shows a positive response. The M→L substitution concerns two apolar amino-acids. The K→D substitution changes a positive with a negative charged amino-acid, but these two amino-acids are then hydrophilic and so do not produce a detrimental α-helix coil in the structure. This *in silico* analysis provides support for how the hIFNα2 antibody assay may recognize *Rousettus* and *Pteropus* IFNα protein.

### IFNα Proteins Are Constitutively Elevated in Plasma of Bat Species

**Figure 2:**
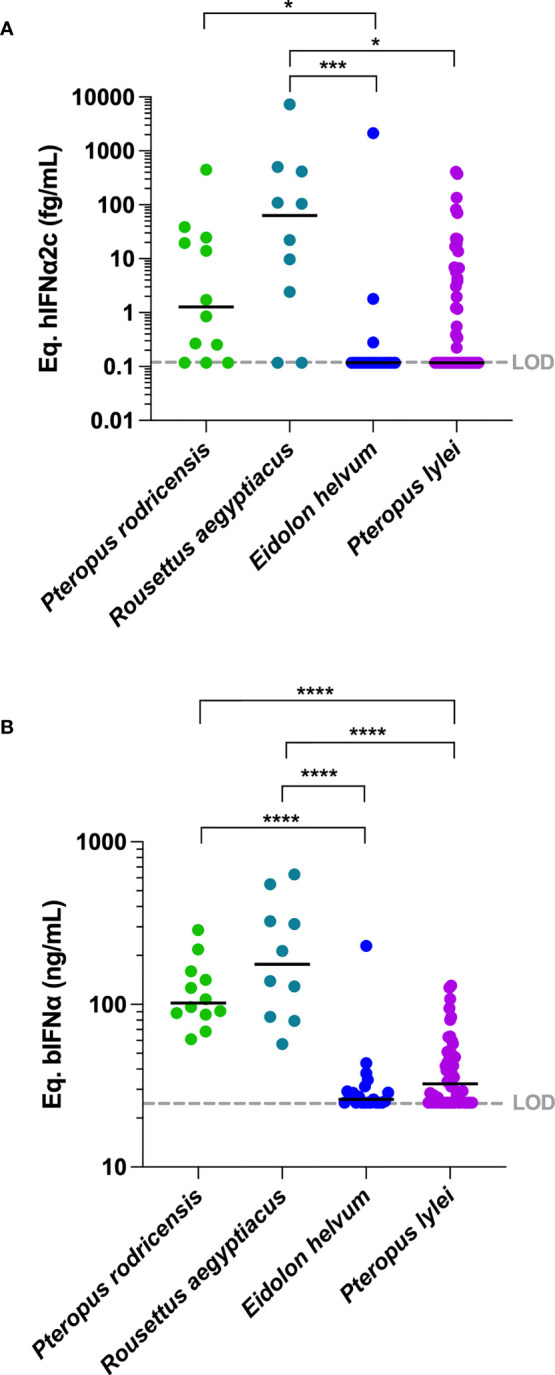
IFNα protein concentrations in plasma of four bat species. IFNα concentrations measured in plasma from four bat species expressed **(A)** as hIFNα2c or **(B)** as bIFNα equivalent concentrations. To allow comparisons, hIFNα2c equivalent concentrations were performed for healthy humans, humans with systemic lupus erythematosus (SLE) and humans with dengue. LOD is the limit of detection level of the assay. Median represented by black line with individual animals shown by colour coded dots. Kruskal–Wallis test with Dunn’s post testing for multiple comparisons was used, *p < 0.05, ***p < 0.001, ****p < 0.0001. P*. rodricensis* (green, n=12), *R. aegyptiacus* (light blue, n=10), *E. helvum* (dark blue, n=21), *P. lylei* (purple, n=52), healthy humans (light grey, n=20), humans with SLE (grey, n=24) and humans with dengue (black, n=24).

Having validated the assay for its ability to detect bat IFNα, we analyzed plasma samples from 4 bat species sampled from French zoos (**Table 1**) with the digital ELISA assay. Results are presented using the two calibrators; hIFNα2c (**Figure 2A**) and bIFNα (**Figure 2B**). A greater number of samples were above the assay limit of detection using the bat protein calibrator as compared to the human protein, confirming the interest of using a bat specific protein. IFNα protein responses obtained within species were relatively consistent. *Pteropus rodricensis* and *Rousettus aegyptiacus* showed significantly elevated IFNα protein plasma levels as compared to *Eidolon helvum* and *Pteropus lylei*, with both the hIFNα2c (p<0.05) and bIFNα (p<0.0001) calibrators. Notably in *Eidolon helvum* plasma IFNα was largely undetectable with both assays revealing interesting inter-species differences. These bat IFNα levels were elevated compared to healthy humans and were in the same range as those observed in auto-immune disease such as Lupus and acute viral infection with dengue virus (**Figure 2A**). When available, we assessed whether age, sex, or presence of clinical alterations (**Table S1**) were associated with IFNα protein levels in all species but found no significant associations (**Figure S3**). We also tested for presence of corona viruses in the blood but found no evidence (data not shown). These results support the hypothesis that certain bat species have physiologyical levels of circulating IFNα protein in healthy conditions.

### IFNα mRNA Are Constitutively Expressed in Bat Leukocytes

**Figure 3:**
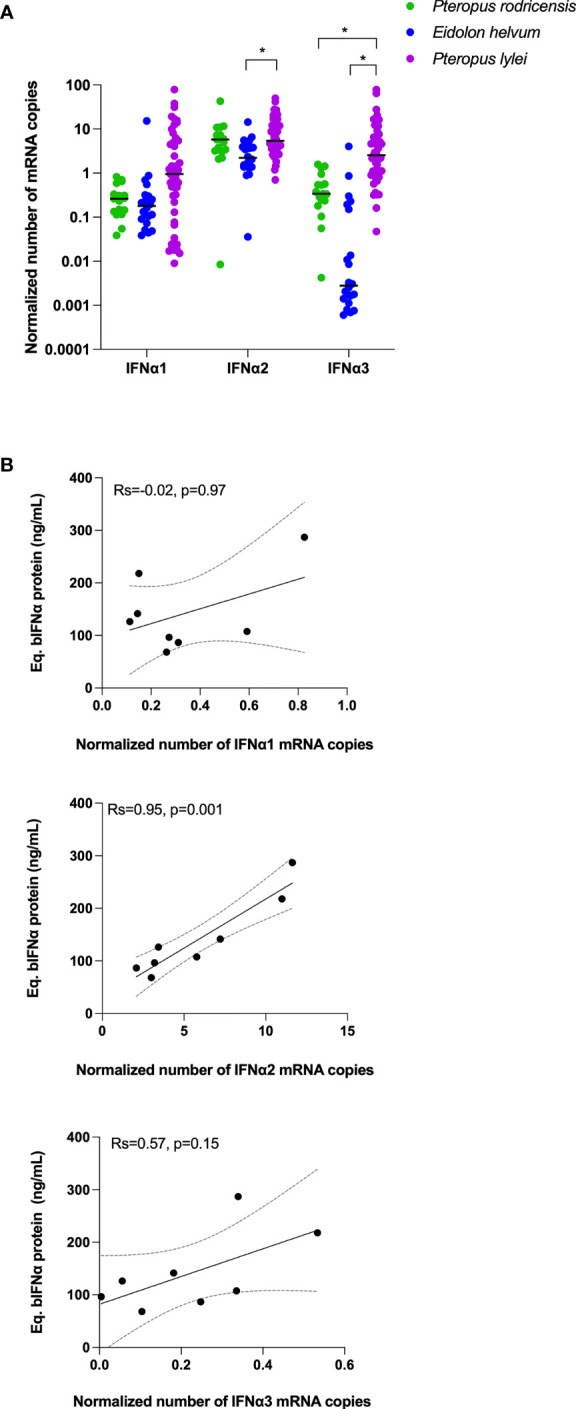
IFNα mRNA levels in bats and mRNA-protein correlations. **(A)** Number of copies measured in whole blood for IFNα1, IFNα2 and IFNα3 mRNAs as normalized to GAPDH in three bat species. Probes used here were not suitable for *Rousettus aegyptiacus*. Median represented by black line with individual animals shown by colour coded dots. Kruskal–Wallis test with Dunn’s post testing for multiple comparisons was used. *p<0.05. **(B)** Correlation plots between the Eq. bIFNα protein concentration obtained using the hIFNα2 digital ELISA assay and the GAPDH-normalized number of IFNα1, IFNα2 and IFNα3 mRNA copies. Spearman method is used for correlation analysis with Spearman’s Rank Correlation Coefficient R (Rs) and p values reported (n=8).

The constitutive mRNA expression of bat IFNα genes has been previously described for *Rousettus aegyptiacus* (33) and *Pteropus Alecto* (30). To test whether the protein plasma levels we observed were linked with leukocyte mRNA expression, and to extend these observations to *Eidolon helvum*, *Pteropus rodricensis* and *Pteropus lylei*, we quantified gene expression of IFNα1, IFNα2 and IFNα3 in our cohort using RT-qPCR and normalizing the results using GAPDH mRNA (**Figure 3A**). Results from *Rousettus aegyptiacus* were negative, perhaps explained by a lack of specificity of the primers utilized. In the other bat species examined the number of IFNα mRNA copies were globally similar to GAPDH, supporting that IFNα species are constitutively expressed at the mRNA level. The IFNα2 subtype mRNA was the most expressed, while IFNα3 was the most variable between species. *Eidolon helvum* had the lowest IFNα mRNA levels, reflecting the absence of detectable protein in this species. *Pteropus lylei* had overall the highest IFNα mRNA levels. Significant differences (p<0.05) were observed between *Eidolon helvum* and *Pteropus lylei* for IFNα2 and IFNα3, and between *Pteropus rodricensis* and *Pteropus lylei* for IFNα3. This also indicates that high levels of variation could be observed within a same genus, and also within the same species.

### IFNα mRNA Expression and Protein Production Could Be Linked Within Each Species

While *Eidolon helvum* showed lower mRNA and protein levels as compared to the other species, and *Pteropus rodricensis* medium mRNA levels and a high protein level, *Pteropus lylei* showed the highest RNA and the lowest protein levels (**Figure 2A, B** and **Figure 3A**). Therefore, we observed no overall direct link between IFNα mRNA expression and protein plasma concentrations across all species examined. To directly explore this hypothesis, we tested the correlations for each gene and protein for *Pteropus rodricensis* samples where sufficient detectable measurements were available for both parameters. In this relatively small sample size (n=8 for paired samples) we observed a strong statistically significant correlation (Rs=0.952, p=0.001) between IFNα2 RNA and protein levels (**Figure 3B**). No correlation was observed with IFNα1 and IFNα3 genes (**Figure 3B**). This result may reflect that the ELISA assay is designed for the IFNα2 subtype.

## Discussion

Type I interferons trigger the downstream activation of hundreds of critical genes as part of the anti-viral immune response. Because of this potency, IFN proteins are secreted at relatively low concentrations as compared to other major cytokines, and multiple regulatory mechanisms exist to control their effects (38). Potential negative consequences of over activation of interferon responses is reflected by their direct implication in multiple autoimmune conditions such as lupus and interferonopathies (39). Furthermore, long-term treatment of patients (ie chronic HCV patients) with type I IFN based therapies can induce serious side effects including depression (40).

Because of these potential negative effects of IFN signaling, recent studies that reported constitutive IFN gene expression in healthy bats were unexpected. However, it provided strong evidence for how bats may potentially live healthily with multiple viral species that are pathological to other mammals including humans. Nevertheless, it raised many additional questions, namely the one which we addressed in our study as to whether IFN protein is also constitutively elevated in bats. This question is not so trivial to address for multiple reasons. IFN proteins have been challenging to directly quantify due to their low physiological concentrations in biological samples, and most studies have utilized proxy readouts such as interferon stimulated gene (ISG) expression or cytopathic assays. The development of digital ELISA such as Simoa overcame these challenges as we demonstrated by the measurement of all 13 IFNα human subtypes, and more recently specifically IFNα2 in multiple human cohorts (31, 32, 41). However, additional challenges exist for the study of interferon in non-human species, in particular bats, most notably the lack of specific reagents, in particular monoclonal antibodies which are required for ELISA technologies. We tested the hypothesis that attomolar digital ELISA sensitivity combined with species cross-reactivity would enable the quantification of bat IFNα protein. After assay validation on virus stimulated bat cell cultures, we further modified the assay with a recombinant bat protein as the standard calibrator. Applying this assay to 4 species of bats conclusively showed constitutive expression of IFNα protein in the circulation of healthy animals. While we cannot yet make a direct comparison, these levels are significantly higher as compared to healthy humans (32), and comparable to those of patients with Lupus or acute infection with dengue.

Our study contains some inherent weaknesses. Because 75% of our bat mRNA samples were of the *Pteropus* genus, we used primers validated and published for *Pteropus alecto* (30)specifying the 3 IFNα1, IFNα2 and IFNα3 subtypes, among which we had a Simoa digital ELISA recognizing human IFNα2, and attempted to use them in species known to have widely different IFNα genes. Accordingly, not having specific primers for each bat species, the primer efficiency to detect IFNα mRNAs could vary and explain the differences observed between species at the mRNA level. Particularly, *Rousettus aegyptiacus* shows no IFNα mRNA expression in this study. The *Rousettus aegyptiacus* genome has been published in total and annotated (42) from which, 12 IFNα genes numbered from 1 to 12 are described. To design primers comparable to those described in Zhou et al. (30), the *Rousettus aegyptiacus* genes corresponding to each *Pteropus alecto* IFNα1, IFNα2 and IFNα3 genes must be identified from Pavlovich et al. (42) who published the genome in NCBI. Analysis of this data shows that the species harbors only two IFNα1-like, one IFNα17-like, and one IFNα4-like genes. To find these corresponding genes in *Rousettus aegyptiacus*, we have scanned the database using BLAST and the three *Pteropus alecto* IFNα1, IFNα2, and IFNα3 sequences. This returns the same *Rousettus aegyptiacus* IFNα4-like gene as the best fit. These comparisons highlight differences in the IFNα genes of the two species, making design of primers to allow a direct comparison challenging, and potentially explaining why our primers did not amplify in the *Rousettus aegyptiacus* samples. Omatsu *et al.* showed that IFNα mRNA was not expressed in *Rousettus leschenaulti* kidney cells and *Tadarida brasiliensis* lung epithelial cells without induction (33), but these cells are very different to circulating immune cells. *Rousettus aegyptiacus* expressed the highest IFNα protein concentrations in our study. Another BLAST search of the IFNα sequence published by Omatsu et al. (33) in the entire *Rousettus aegyptiacus* genome obtained by Pavlovich et al. (42) returns 13 sequences with more than 88% homology (10 >94%) at the amino-acid level. Interestingly, all of these 13 IFNα proteins have the same LLDED epitope that we believe is recognized by our Simoa IFNα2 assay. Therefore, one hypothesis could be that because of their high number, detected IFNα protein levels could be high even if the individual mRNA levels are low and undetectable. As for mRNA considerations, the affinity of the hIFNα2 Simoa assay antibodies for the bat IFNα proteins could be different from one species to another. This could also explain the differences observed between species at the protein level. Moreover, IFNα protein concentrations were calculated using human IFNα2c or *Rousettus aegyptiacus* IFNα protein (bIFNα) produced in *E. coli* as calibrators. The bIFNα is not the main component of the raw soluble *E. coli* extract that is used as the calibrator. As an ELISA technique, this assay has no reactivity for *E. coli* proteins, just for IFNα protein which means purity is not a necessity. However, to quantify IFNα in bat samples, the concentration of the recombinant bat IFNα in this lysate must be obtained. This was done by SDS-Page densitometry after identification of the bat IFNα recombinant protein using Western-Blot. The use of these two human and bat calibrators produces results respectively in the fg/mL and the ng/mL ranges. The order of magnitude provided by the pure human protein is probably too low because of the higher sensitivity of our ELISA assay for human than bat proteins. Conversely the order of magnitude of bat IFNα concentrations given by the crude bIFNα extract is probably too high because of the incorrect folding of the bIFNα protein produced in *E. coli* strains. This is supported by the observations that the bIFNα protein is mainly expressed in the insoluble fraction even at 16°C (**Figure S1F**), that the protein failed to renature after urea purification (**Figure S1E**), and that the viral inactivation protocol had a greater effect on the hIFNα2 assay response to bIFNα (>1Log) than to hIFNα2c (**Figure S4**). These observations suggest a greater insolubility of the bat protein. Additional improvements of the assay could be envisioned such as production of a purer and better folded recombinant protein in mammalian cells, and eventually the production of a bat specific monoclonal antibody against this protein.

Despite these technical limitations, we were able to show elevated levels of plasma IFNα protein in certain bat species, which also correlated with expression levels of the IFNα2 gene. While additional confirmatory experiments will be required, the inter-species differences in plasma IFNα protein is an interesting observation. It would also be interesting in future studies to assess whether these IFNα protein differences have an impact on viral levels and diversity within the different bat species. Lastly, our results raise additional new questions on the nature of bat physiology, in particular how the constitutively activated type I IFN response is maintained in bats without resulting in pathological conditions such as those observed in human autoimmune disease. Chronic IFN activation, in particular during growing and development phases, can have significant neurological effects as observed in interferonopathies such as STING mutation patients (43). Finally given the important role of type I interferon for protection to infection with SARS-CoV-2 (36, 44), and the potential role bats have played in seeding the COVID-19 pandemic (20) understanding this host-virus relationship could have major implications for pandemic preparedness. In summary improved knowledge on the special nature of bat IFN regulation could have major implications for our basic understanding of IFN biology, its continued use as a therapeutic, and our capacity to prepare for viral pandemics.

## Data Availability Statement

The original contributions presented in the study are included in the article/**Supplementary Material**. Further inquiries can be directed to the corresponding authors.

## Ethics Statement

The studies involving human participants were from previously published studies where ethical committee details are described. The patients/participants provided their written informed consent to participate in this study. Ethical review and approval was not required for the animal study because the bat specimens included in this study are not wild but obtained from zoos where they have been maintained for several generations. Animals were not sampled for the purpose of this study. Instead, blood was taken during routine clinical examination by authorized veterinarians for which no specific ethical requirements were required. However, for biosafety precautions, samples were handled in a P2 biosafety level laboratory where they were treated with a viral inactivation protocol before use as described above.

## Author Contributions

VB and MB: Data generation and curation, formal analysis, methodology, validation, visualization, writing—original draft, and writing—review and editing. SP: Data curation, methodology, and validation. AL, TP, and RW: Resources, methodology, project administration, and validation. DD and DR: Conceptualization, project administration, resources, supervision, validation, roles/writing—original draft, and writing—review and editing. All authors contributed to the article and approved the submitted version.

## Funding

This work was funded by the Agence Nationale pour la Recherche (ANR), grant number CE17001002.

## Conflict of Interest

The authors declare that the research was conducted in the absence of any commercial or financial relationships that could be construed as a potential conflict of interest.

## Publisher’s Note

All claims expressed in this article are solely those of the authors and do not necessarily represent those of their affiliated organizations, or those of the publisher, the editors and the reviewers. Any product that may be evaluated in this article, or claim that may be made by its manufacturer, is not guaranteed or endorsed by the publisher.

## Supplementary Material

Supplementary Figure 1 Production and purification of the bIFNα calibrator. **(A)** Amino-acid sequence of the *Rousettus aegyptiacus* IFNα protein as described in (33). The signal peptide is highlighted in blue. **(B)** Amino-acid sequence of the bIFNα protein as designed for production. The sequence corresponding to the 6His tag for purification, spacers and the TEV cleavage site to remove the tag is highlighted in red. **(C)** Map of the pT7 expression plasmid used for bIFNα production in *Escherichia coli.* **(D)** SDS-Page profile obtained before IPTG induction (Ø) and after induction at 16, 30 or 37°C in the soluble or insoluble extract of *Escherichia coli* cells transformed to produce bIFNα. Molecular weights (MW) are indicated in kDa (left panel). Western-Blot profile obtained from the soluble fraction after induction at 16°C (right panel). After SDS-Page migration, proteins were transferred on a nitrocellulose membrane then incubated with the hIFNα2 assay detection antibody. Streptavidin-conjugated horseradish peroxydase was added to the membrane and signal was revealed using chemiluminescence. **(E)** SDS-Page profile for each bIFNα purification step from the soluble fraction (left panel) after IPTG induction at 16°C. Column load (IN), column flow-through (FT), washes (W1, W2 and W3) and elution fractions (E1 to E9). The middle panel shows the SDS-Page profile for the pools of eluted fractions. The red arrow indicates the bIFNα protein at its right molecular weight. **(F)** SDS-Page profile for each bIFNα purification step from the insoluble fraction (left panel) after IPTG induction at 16°C as previously described. Right panel shows SDS-Page profile for each renaturation step of the pool of eluted fractions obtained from the insoluble *E. coli* extract: 4M, then 2M, then 1M urea and finally without urea (ØM), the ultimate step where the protein is lost. Red arrows indicate the bIFNα protein at his right molecular weight.

Supplementary Figure 2 Bat IFNα proteins have a similar hIFNα2 assay epitope to human IFNα proteins. Protein sequence alignment obtained for human IFNα1, IFNα2, IFNα16 and IFNα17, for *Pteropus alecto* IFNα1, IFNα2 and IFNα3, for *Rousettus aegyptiacus* IFNα, and for mouse IFNα1, IFNα3, IFNα4, IFNα11 and IFNα13 using the CLUSTALW software from position 90 to 130 referred to hIFNα2. The red rectangle highlights the suspected epitope recognized by the antibodies of the hIFNα2 assay.

Supplementary Figure 3 Age, sex and clinical alterations were not associated with IFNα protein levels. **(A)** Correlation plot between age of all studied bat specimens and IFNα protein levels expressed as bIFNα equivalent concentration. Spearman method is used. **(B)** IFNα protein levels expressed as bIFNα equivalent concentration for female and male groups, all species combined. Median represented by black line. Mann-Whitney test is used, ns: p>0.05. **(C)** IFNα protein levels expressed as bIFNα equivalent concentration for no clinical signs and health alterations groups, all species combined. Health alterations correspond to mild symptoms such as pale wings or chest alopecia but excluding pregnant or nursing bats. Details are described in **Table S1**. Median represented by black line. Mann-Whitney test is used, ns: p>0.05.

Supplementary Figure 4 Viral inactivation decreases the affinity of the assay for bIFNα but not hIFNα2c. hIFNα2 assay response (AEB) as a function of IFNα concentrations for hIFNα2c and bIFNα untreated or after viral inactivation.

Supplementary Table 1 All data available for each sample. Species, gender, GAPDH-normalized number of IFNα1, IFNα2 and IFNα3 mRNA copies, interferon α concentrations expressed as hIFNα2c or bIFNα equivalent concentrations and notes available for each sample.

Supplementary Table 2 Primers and probes sequences. List of primers and probes used in RT-qPCR for bat IFNα1, IFNα2, IFNα3 and GADPH, based on published data from Zhou et al. (30) for the bat IFN genes described for *P. alecto* and designed for this study for GADPH, from PrimerBLAST and available datas for GADPH gene in *P. alecto* and *R. aegyptiacus* species.

## Abbreviations

AEB, average enzyme per bead; HAU, hemagglutinating units; IFN, interferon; LOD, limit of detection.
