# Supplementary figures and images for: Constitutive IFNα Protein Production in Bats
Source: Front Immunol. 2021 Nov 1;12:735866. doi: 10.3389/fimmu.2021.735866 (PMC8591296; doi:10.3389/fimmu.2021.735866)

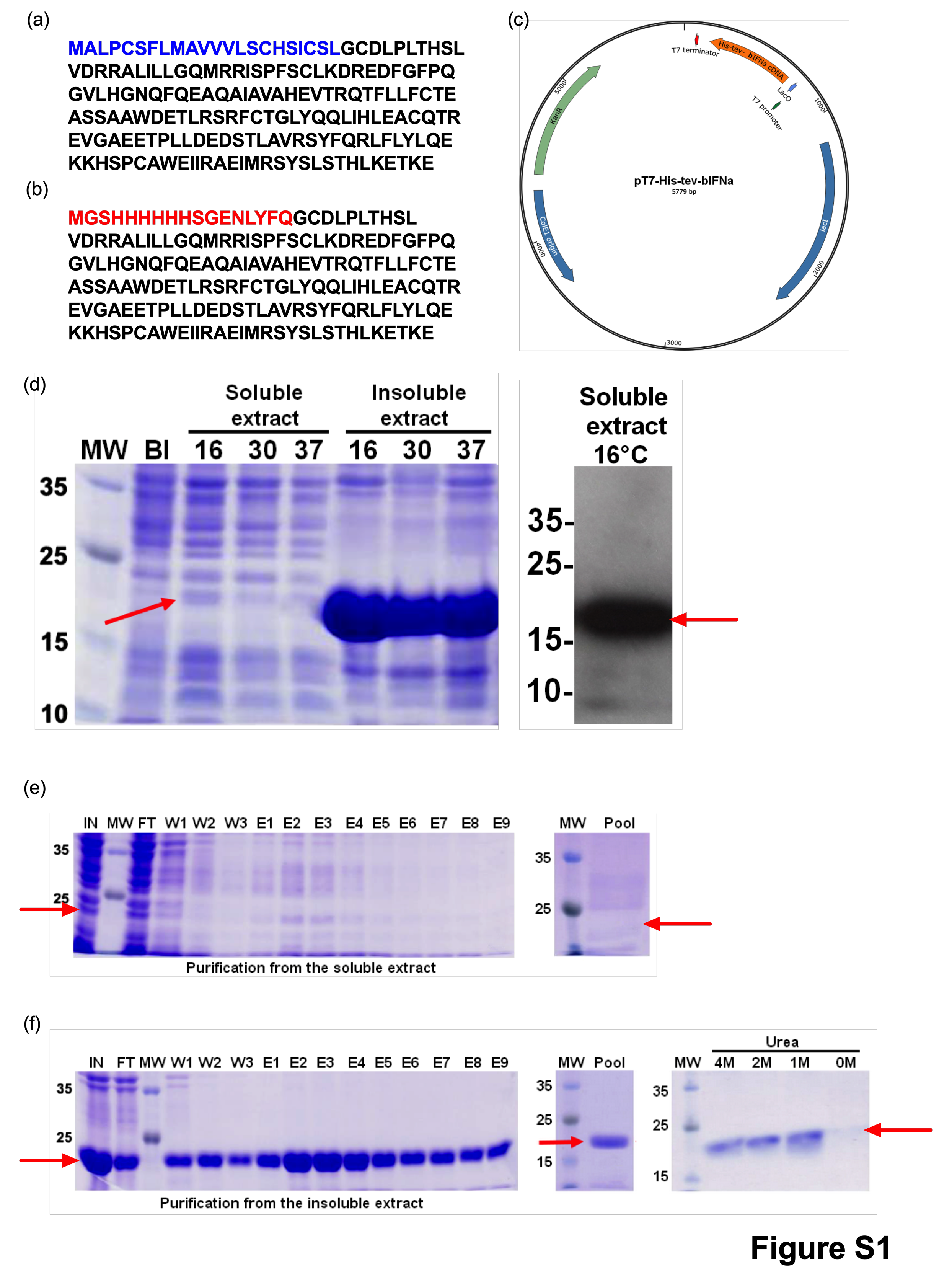

Supplement: Supplementary Figure 1 — Production and purification of the bIFNα calibrator. (A) Amino-acid sequence of the Rousettus aegyptiacus IFNα protein as described in (33). The signal peptide is highlighted in blue. (B) Amino-acid sequence of the bIFNα protein as designed for production. The sequence corresponding to the 6His tag for purification, spacers and the TEV cleavage site to remove the tag is highlighted in red. (C) Map of the pT7 expression plasmid used for bIFNα production in Escherichia coli. (D) SDS-Page profile obtained before IPTG induction (Ø) and after induction at 16, 30 or 37°C in the soluble or insoluble extract of Escherichia coli cells transformed to produce bIFNα. Molecular weights (MW) are indicated in kDa (left panel). Western-Blot profile obtained from the soluble fraction after induction at 16°C (right panel). After SDS-Page migration, proteins were transferred on a nitrocellulose membrane then incubated with the hIFNα2 assay detection antibody. Streptavidin-conjugated horseradish peroxydase was added to the membrane and signal was revealed using chemiluminescence. (E) SDS-Page profile for each bIFNα purification step from the soluble fraction (left panel) after IPTG induction at 16°C. Column load (IN), column flow-through (FT), washes (W1, W2 and W3) and elution fractions (E1 to E9). The middle panel shows the SDS-Page profile for the pools of eluted fractions. The red arrow indicates the bIFNα protein at its right molecular weight. (F) SDS-Page profile for each bIFNα purification step from the insoluble fraction (left panel) after IPTG induction at 16°C as previously described. Right panel shows SDS-Page profile for each renaturation step of the pool of eluted fractions obtained from the insoluble E. coli extract: 4M, then 2M, then 1M urea and finally without urea (ØM), the ultimate step where the protein is lost. Red arrows indicate the bIFNα protein at his right molecular weight. [file Image_1.tiff]

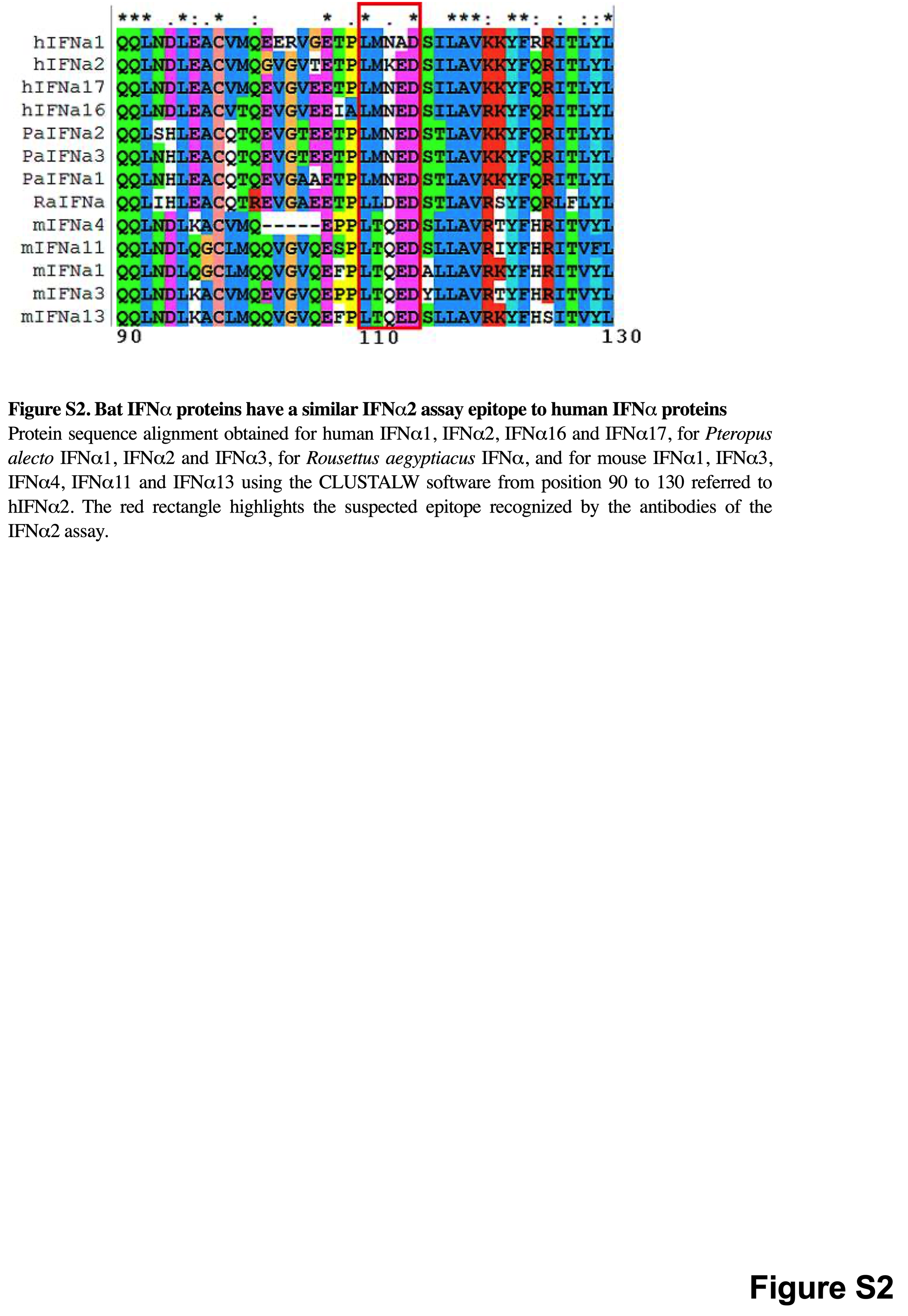

Supplement: Supplementary Figure 2 — Bat IFNα proteins have a similar hIFNα2 assay epitope to human IFNα proteins. Protein sequence alignment obtained for human IFNα1, IFNα2, IFNα16 and IFNα17, for Pteropus alecto IFNα1, IFNα2 and IFNα3, for Rousettus aegyptiacus IFNα, and for mouse IFNα1, IFNα3, IFNα4, IFNα11 and IFNα13 using the CLUSTALW software from position 90 to 130 referred to hIFNα2. The red rectangle highlights the suspected epitope recognized by the antibodies of the hIFNα2 assay. [file Image_2.tiff]

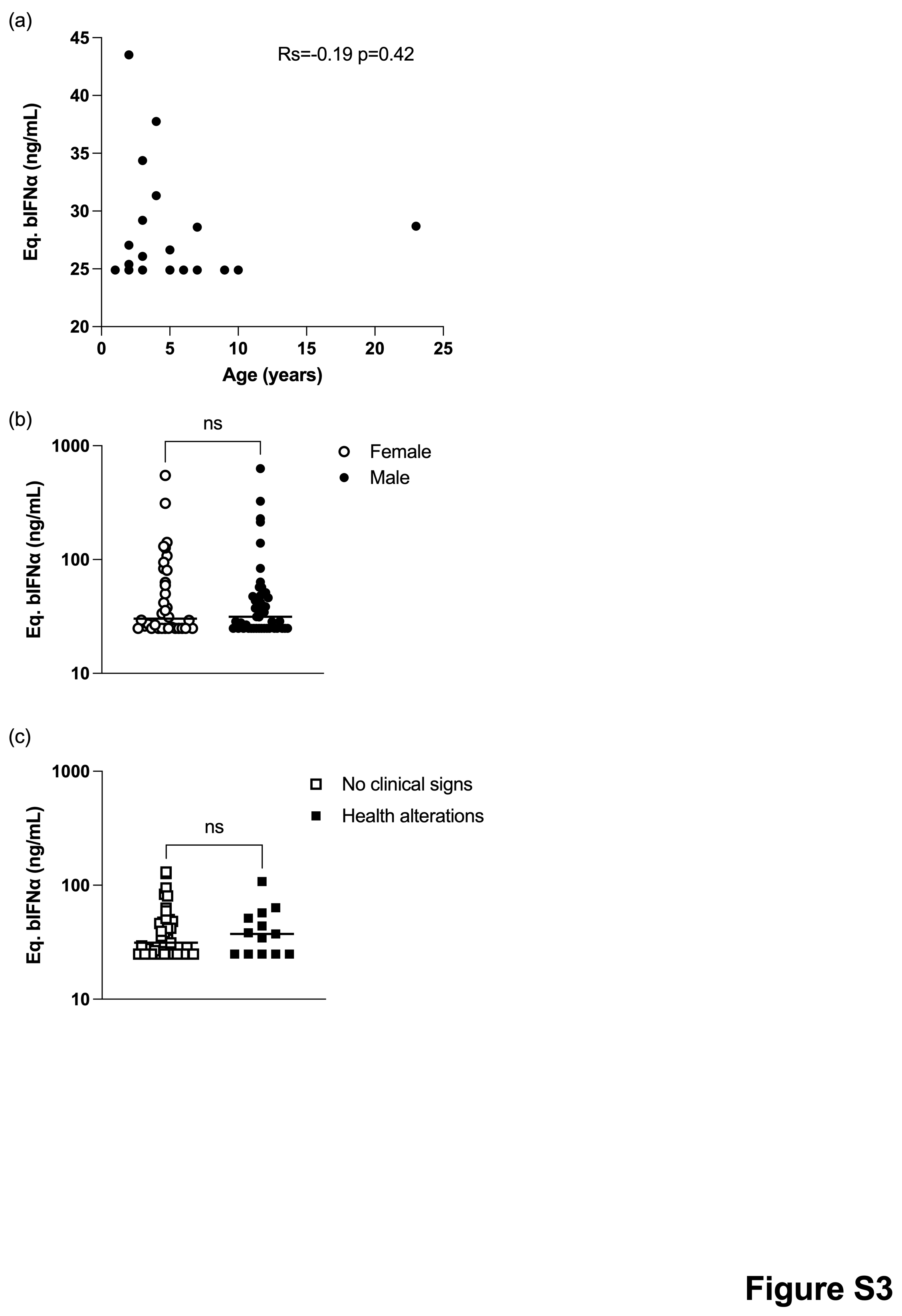

Supplement: Supplementary Figure 3 — Age, sex and clinical alterations were not associated with IFNα protein levels. (A) Correlation plot between age of all studied bat specimens and IFNα protein levels expressed as bIFNα equivalent concentration. Spearman method is used. (B) IFNα protein levels expressed as bIFNα equivalent concentration for female and male groups, all species combined. Median represented by black line. Mann-Whitney test is used, ns: p>0.05. (C) IFNα protein levels expressed as bIFNα equivalent concentration for no clinical signs and health alterations groups, all species combined. Health alterations correspond to mild symptoms such as pale wings or chest alopecia but excluding pregnant or nursing bats. Details are described in Table S1 . Median represented by black line. Mann-Whitney test is used, ns: p>0.05. [file Image_3.tiff]

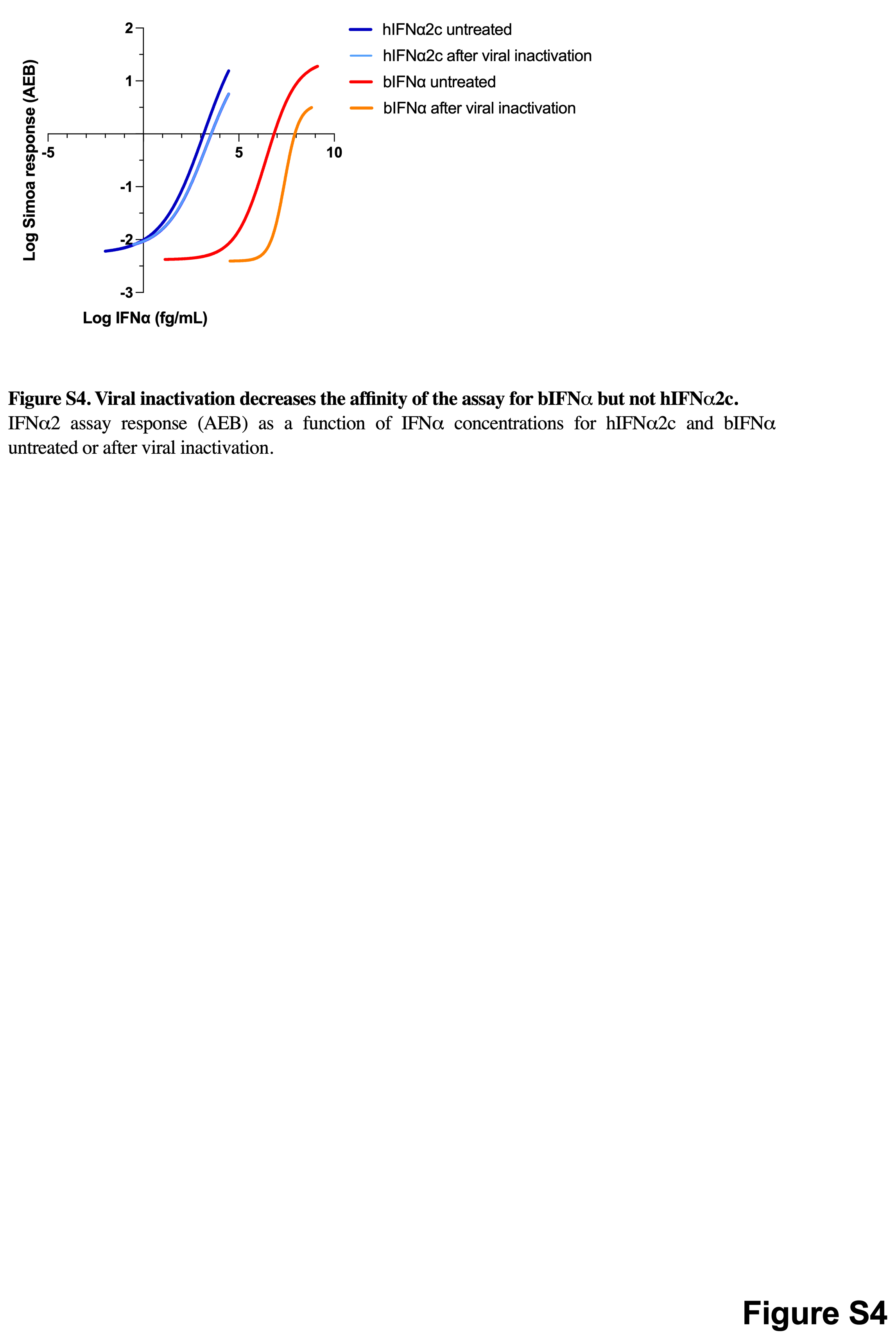

Supplement: Supplementary Figure 4 — Viral inactivation decreases the affinity of the assay for bIFNα but not hIFNα2c. hIFNα2 assay response (AEB) as a function of IFNα concentrations for hIFNα2c and bIFNα untreated or after viral inactivation. [file Image_4.tiff]
